# Comparing Watchful Waiting Approach vs. Antibiotic Therapy in Children with Nonsevere Acute Otitis Media: A Randomized Clinical Trial

**DOI:** 10.1155/2021/5515467

**Published:** 2021-05-27

**Authors:** Leila Shahbaznejad, Ensieh Talaei, Fatemeh Hosseinzadeh, Baraneh Masoumi, Shaghayegh Rezai, Mohammad Sadegh Rezai

**Affiliations:** ^1^Pediatric Infectious Diseases Research Center, Communicable Diseases Institute, Mazandaran University of Medical Sciences, Sari, Iran; ^2^Department of Microbiology and Virology, Mashhad University of Medical Sciences, Mashhad, Iran

## Abstract

**Objective:**

To compare both approaches for the treatment of nonsevere acute otitis media (AOM) in Iran.

**Methods:**

This randomized clinical trial was performed at a pediatric infectious diseases clinic in Buali tertiary hospital in Sari, north of Iran, from 2016 to 2018. All participants in this study were previously healthy children with AOM diagnosis, who were 6 months to 6 years old. The patients were randomly assigned into two groups: the intervention (80 mg/kg/day amoxicillin for 7-10 days) and the control group (watchful waiting approach). AOM recovery and adverse drug reactions were evaluated after 72 hours, and the patients were followed for the frequency of AOM and middle ear effusion 1 and 3 months' postintervention.

**Results:**

A total of 396 children have participated in this study. AOM recovery was significantly different in the two groups (73% vs. 44% in the intervention and control groups, respectively). Recurrence of AOM and middle ear effusion (MEE) persistence, one month following the intervention, have not shown any significant differences between the two groups. However, the AOM recurrence between 1 and 3 months was more frequent in the control group. The frequency of diarrhea was also higher in the intervention group compared to the control but no significant difference was found between the two groups regarding vomiting and skin rash.

**Conclusion:**

The faster recovery from AOM is achieved when an antibiotic treatment regimen is applied, although the risk of potential side effects should be considered.

## 1. Introduction

Acute otitis media (AOM) is one of the most common childhood diseases. Nearly 75% of children experience at least one episode of ear infection before starting their school [1, 2]. AOM is one of the leading causes of healthcare visits in many countries. The worldwide AOM rate is estimated to be about 10.85% (709 million cases) each year, from which 51% of them are under the age of five [3].

Age, gender, race, genetics, socioeconomic status, neonatal feeding, smoke exposure, day care attendance, season, and the midline facial defects are the most common factors affecting the rate of AOM infections in children [1, 3, 4]. Otitis media is more common in boys aged 6 to 20 months [5]. Socioeconomic status can result in many risk factors associated with AOM. Children from lower socioeconomic status may be at more risk of smoke exposure, crowded day care centers, poorer living condition, bottle-feeding, and more viruses and bacterial pathogens [6].

Otitis media more frequently occurs during the cold seasons, due to the increased number of upper respiratory tract infections [7]. Viruses are the most common cause of AOM infections. In addition, some bacteria can cause AOM as well such as *Streptococcus pneumonia*, nontypeable *Haemophilus influenza*, and *Moraxella catarrhalis*. Other pathogens that are less frequently associated with AOM are *Streptococcus type A*, *staphylococcus aureus*, and Gram-negative organisms which are more commonly seen in neonates [8, 9].

Currently, most guidelines suggest two methods of treatment for AOM [10]. The treatment options consist of the use of antibiotics and the “watchful waiting” approach, which was first suggested by the American Academy of Pediatrics (AAP) and American Academy of Family Physicians (AAFP) in 2004. The watchful waiting approach was introduced due to the emergence of antibiotic resistance. The guideline was then modified in 2013 with more emphasis on the importance of accurate diagnosis of AOM [11]. According to the recent guideline, the watchful waiting approach can be used in healthy children with 6-23 months of age who have mild symptoms with appropriately diagnosed unilateral AOM or children ≥2 years old with bilateral or unilateral AOM or children who do not fully meet the diagnostic criteria [12]. The watchful waiting approach can be used for 48 hours if the follow-up is assured. Children ≥6 months with a bulging tympanic membrane, fever (≥39°C) and moderate to severe systemic illness, who have severe otalgia, or have already been significantly ill for ≥48 hours and children <2 years with bilateral AOM regardless of the additional signs or symptoms should be treated with antimicrobial agents [12, 13]. Studies show that up to 80% of AOM cases resolve spontaneously without antibiotics, and antibiotics may increase the risk of vomiting, diarrhea, and rash and also antibiotic resistance [10, 14].

Results of a study in Sweden showed that pneumococcal conjugate vaccines reduce the incidence of otitis media up to 26% and delay the first episode of otitis media in infants and young children [15]. This could be due to the reduced nasopharyngeal colonization of bacteria by PCVs [16]. In 2010, the FDA approved the 13-valent pneumococcal-conjugated vaccines (PCV13), which has shown to reduce the prevalence of AOM in children under 2 years old. This vaccine has been included in their national vaccination program since [4]. However, pneumococcal vaccination is not still included in the national vaccination program of some countries [17].

Neither conjugated and polysaccharide pneumococcal vaccines nor Hib vaccine are routine as part of the national immunization program in Iran. So, in this study, we aimed to compare the success rate of treatment options, potential side effects, and recurrence of AOM in the observational and antimicrobial groups in Iranian children with AOM, who have not received pneumococcal vaccines as part of their national vaccination program.

## 2. Methodology

### 2.1. Study Design and Interventions

This randomized clinical trial was undertaken in the pediatric infectious diseases clinic at Buali tertiary hospital in Sari, north of Iran, between 2016 and 2018. The eligibility criteria for this study included age (6 months to 6 years), AOM diagnosis (acute onset of fever, erythema of tympanic membrane, and middle ear effusion), and onset of the symptoms within 48 hours prior to visiting the physician. Children with severe AOM (fever ≥ 39°C, moderate to severe irritability, and otalgia), otorrhea, concurrent conjunctivitis, underlying diseases such as immunodeficiency, and history of penicillin allergy were excluded from the study.

According to the AAP guideline 2013, patients included in the inclusion criteria were randomly assigned into two groups, the intervention group and the control group [11].

### 2.2. Intervention

The intervention group received a high dose (80 mg/kg/day) of amoxicillin suspension made by Farabi pharmaceutics with the brand name of “Faramox BD” for 7-10 days divided into two doses. For the control group, the “watchful waiting” approach and monitoring were performed. In case of pain and fever, acetaminophen or ibuprophen was prescribed for patients of both groups.

### 2.3. Measures

Clinical symptoms and adverse drug reactions including fever, otalgia, irritability, poor feeding, seizure, vomiting, diarrhea, coryza, cough, pharyngitis, nasal congestion, and allergic reactions were recorded in both groups at the first and fourth days of intervention. Severe complications of AOM such as mastoiditis, petrositis, meningitis, and otorrhea were also evaluated during the study. AOM recovery was defined as fever, irritability, and otalgia elimination after 72 hours.

The outcome measures included recurrent AOM and middle ear effusion (MEE) after one and three months following the intervention. MEE was defined as otorrhea, loss of tympanic membrane mobility, air–fluid level or bubbles behind tympanic membrane, and tympanic edema. AOM was defined as fever in addition to middle ear inflammation and effusion.

### 2.4. Randomization and Mmasking

The patients were randomly divided into two groups, the intervention and control groups, by simple randomization method, and physicians were given a table of precoded numbers and patients enrolled the study in order of table numbers. The total sample size consisted of 400 patients, 200 participants per group. Neither the participants nor the evaluators were aware of the randomization process or group allocation. After obtaining the written informed consent, amoxicillin suspension was given to the parents of the intervention group. So, this study was not blinded.

### 2.5. Ethical Considerations

The ethics committee of Mazandaran University of Medical Sciences approved the study protocol (Code: IR.MAZUMS.REC.1395.2489), and it was registered in the Iranian Registry of Clinical Trials (Code: IRCT20111224008507N2). A written informed consent was obtained from all parents of children prior to enrolment. The trial was conducted in accordance with the principles of the Declaration of Helsinki.

### 2.6. Statistical Analyses

The collected data were analyzed using the 2011 Statistical Package for the Social Sciences (SPSS; IBM, Armonk, New York) software for Windows, version 20. Chi-square, Fisher's exact, and McNemar tests were used to compare the variables before and after the intervention, and the *P* value of less than 0.05 was considered being statistically significant.

## 3. Results

**Figure 1:**
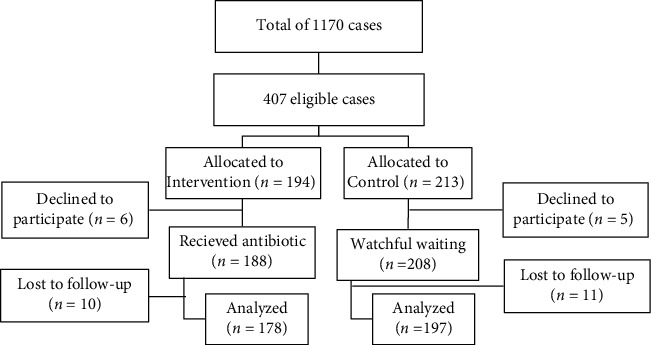
Flow chart of the study allocation and follow-up.

A total of 407 children were enrolled in the study (Figure 1). From these patients, 11 cases (6 patients from the case group and 5 from the control) withdrew due to reluctance. 396 children (188 patients in the intervention group, mean age: 29.05 ± 16.6 months, and 208 ones in control group, mean age: 28.88 ± 15.9 months: *P* > 0.05) have participated in this study, from which 189 children (47.82%) aged 6-23 months and the rest aged 2-6 years.

No significant differences were found between the two groups regarding the symptoms, clinical findings, types of care, feeding, smoke exposure, antibiotic consumption in the last 4 weeks, and vaccination history (Table 1). At the baseline, the most common symptoms or clinical findings in all participants were fever (342 cases, 86.36%), otalgia (200 cases, 50.50%), and irritability (172 cases, 43.43%). Frequencies of the unilateral or bilateral erythema and/or effusion were not significantly different in the two groups (*P* > 0.05).

Three days after the intervention, most of the complaints disappeared in both groups, but the improvements were more significant in the antibiotic group. 28 (15%) patients from the intervention group and 59 (28%) patients from the control group still had otalgia (*P* < 0.01). 35(19%) of the interventions and 92(44%) of the controls had a fever (*P* < 0.01), and 19 (10%) of the interventions and 40 (19%) of the controls had irritability (*P* < 0.05) (Table 2). Overall, AOM recovery was seen in 137 (73%) and 91 (44%) of the patients from the antibiotic-treated group and the control group, respectively (*P* < 0.01).

Furthermore, a significant patient improvement from AOM was observed in those with unilateral or bilateral erythema of tympanic membrane and/or middle ear effusion in children younger or older than 2 years old regardless of their exposure to antibiotic therapy 4 weeks prior to study participation.  Table 3 shows other factors attributed to the improvement of AOM in detail.

Severe complications due to the AOM infections such as meningitis, acute mastoiditis, and petrositis were not seen, but eardrum perforation was reported in two patients from the intervention group and one from the control group (*P* > 0.05) (Table 2).

From a total of 396 patients, 375 cases were followed 1 and 3 months after the intervention. The parents were asked about the recurrence of AOM and MEE symptoms. At the one-month follow-up, no differences were found between the two groups in terms of AOM recurrences. However, observation from the first and three-month follow-up showed lower AOM episodes in the antibiotic-treated group (*P* < 0.05) (Table 4). There was no difference in the recurrence of AOM in both groups based on the history of allergy, asthma, breastfeeding, child care type, smoke exposure, and erythema of tympanic membrane or MEE at the beginning of the study (Table 5). MEE frequency decreased during the time, and it was not significantly different in any of the groups (Table 4).

Drug side effects occurred in 20 (11%) of the intervention and 6 (3%) of the control group patients (Table 4). Vomiting, skin rash, and other side effects were equally seen in both groups but diarrhea was more frequent in the antibiotic-treated group; 13 (7%) in the intervention versus 5 (2%) in the control group (*P* < 0.05).

## 4. Discussion

This study is a randomized controlled trial for the treatment of acute otitis media in children 6 months to 6 years of age. Two different therapeutic approaches are used in the management of AOM in children. According to the recent guidelines, the AOM recovery rate may not be much different in patients with or without antibiotic treatment. Therefore, both watchful waiting approach and antibiotic therapy are encouraged where appropriate [13].

According to our study, when antibiotic (high dose of amoxicillin) was prescribed to children with nonsevere AOM aged 6 months to 6 years, the AOM recovery rate was significantly higher compared to the watchful waiting group during the first 72 hours (73% vs. 44%). In other studies, the AOM recovery in the antibiotic receiving groups ranged from 41% to 92.8% [18–21] versus 28% to 84% in the watchful waiting group [18–22]. These differences in the outcomes may be due to participants' age (younger or older than 2 years), type and different doses of antibiotics, i.e., amoxicillin 40 to 80 mg/kg/day, or amoxicillin–clavulanate, matching groups regarding vaccination against influenza, day care attendance, breastfeeding status, history of AOM recurrence, and exposure to smoke. Antimicrobial therapy for AOM has been attributed to be a major factor in the emergence of resistance among otopathogens; hence, current guidelines endorse withholding antibiotics as an option in selected children where appropriate [18–23]. However, it should be considered that other studies have included children who were vaccinated against pneumococcal pathogens, which could have altered the AOM pattern in children. Studies on AOM have shown a 29% rate reduction of otitis media infections in children receiving this vaccine before the age of 24 months [24, 25]. Furthermore, a 40% reduction of healthcare visits for otitis media has also been observed in children who were vaccinated [26, 27]. Only 1.5% of children in our study were vaccinated with the pneumococcal-conjugated vaccine (PCV13). Therefore, due to the limited number of vaccinated participants, we could not evaluate the effect of this vaccine on AOM.

In our study, the use of antibiotics significantly improved the AOM in both age groups of 6 months to 2 years and 2 to 6 years old with or without the use of antibiotic treatment in the last 4 weeks prior to this study.  Table 3 shows the other association between treatment response and different factors. However, the difference in improvement between the antibiotic vs. watchful waiting group was not observed in children that attended day care centers or exposed to smoke. Studies reported that exposure to smoke or attending day care centers are risk factors for AOM in children and may alter host defence against organism or exposed children to more virulent strains [28, 29]. In addition, due to a limited number of patients who received pneumococcal vaccine, we could not have any discussions about the effect of these vaccines on AOM in our study.

In terms of adverse effects, our findings were in coordination with other studies, for example, diarrhea was more prevalent in the antibiotic-treated group, which was self-limited [16–18, 22]. Nausea and vomiting were not significantly different in both groups. Despite the adverse effects of antibiotics, it seems that most of the patients may benefit from antibiotics and these mild side effects can be managed with proper follow-ups. Other side effects, which were reported previously like skin rash or eczema, were not seen in our patients [18]. In our study, no serious AOM complications such as meningitis, acute mastoiditis, or petrositis were observed in both groups but eardrum perforation was reported in two patients from the intervention group and one patient from the control group. Serious complications are rare in AOM patients, and eardrum perforation is the most common complication [30]. Therefore, due to the small sample size, we could not discuss the effect of antibiotics or watchful waiting approach on serious complications. No serious AOM-related adverse effects were reported in either groups of McCormick et al.'s study [22]. However, Hoberman et al. reported one case of acute mastoiditis in the placebo group. They have also reported tympanic perforation in 1% of the patients in the antibiotic group and 5% in the placebo group [19]. Damoiseaux et al. and Tähtinen et al. reported tympanic perforation in 15.3% and 0.6% of the antibiotic group compared to 17% and 3.2% of the control group, respectively [18, 20].

Some of the long-term concerns regarding the management of AOM are the disease recurrence and persistence of MEE. It is not clear how antibiotic use or watchful waiting alters these consequences. In our study, no significant difference was found in the recurrence of AOM or persistence of MEE between two groups one-month postintervention. However, after 3 months, AOM recurrence was statistically higher in the control group. These findings were in coordination with some other studies [18, 22, 24]. Contrary to our findings, Hoberman et al.'s and Le Saux et al.'s studies reported that the recurrence rate of MEE and AOM were insignificantly higher in the control group after one month of treatment [19, 21]. Based on these results, it is concluded that in either short-term (4-6 weeks) or long-term (3 months) follow-ups, the therapeutic choices may not alter the recurrence rate of AOM or MEE. We investigated factors such as the history of allergy or asthma, breastfeeding, child care type, and smoke exposure as effective factors in AOM recurrence or MEE in the two groups but we did not find any differences.

Our study showed that antibiotic therapy is superior in comparison to the observational approach in children who have not received pneumococcal vaccination. Antibiotic treatment is more beneficial in patients who can be monitored despite its adverse effects. The main limitation of our study was the lack of a placebo and not being able to perform a double-blinded study. Due to the infrequency of serious complications of AOM, further multicenter studies with numbered cases may be beneficial. Study on the effect of antibiotic usage in appearing more virulent strains and/or microbial resistance needs long-term studies and experienced laboratories.

## 5. Conclusion

In this study, we have investigated the appropriateness of the watchful waiting approach vs. antibiotic therapy in the management of AOM in a pediatric infectious diseases clinic of a tertiary hospital in Iran. It is concluded from our findings that despite the risk of bacterial resistance and potential side effects of antibiotics, faster symptom improvements are achieved when antibiotics are prescribed in children 6 months to 6 years with nonsevere AOM in our country. It could be attributed to the reason that pneumococcal vaccination is not included in the national vaccination program of Iran.

## Figures and Tables

**Table 1:** Demographic and characteristics of the patients.

		Group		*P* value
		Intervention (*N*: 188)	Control (*N*: 208)	
Gender	Male	104 (55)	95 (46)	>0.05
	Female	84 (45)	113 (54)	
Age group (months)	6-23	92 (49)	97 (47)	>0.05
	>24	96 (51)	111 (53)	
Vaccination	Yes	188 (100)	208 (100)	>0.05
	No	0 (0)	0 (0)	
Pneumococcal vaccination	Yes	3 (1.6)	3 (1.4)	>0.05
	No	185 (98.4)	205 (98.6)	
Antibiotic in last 4 weeks	Yes	109 (58)	136 (65)	>0.05
	No	79 (42)	72 (35)	
History of breastfeeding	Exclusive breastfeeding	128 (68)	136 (66)	>0.05
	Formula-fed	24 (13)	28 (14)	
	Both	36 (19)	41 (20)	
Child care type	Day care center	48 (26)	57 (27)	>0.05
	Babysitter	10 (5)	8 (4)	
	Mother	130 (69)	143 (69)	
Smoke exposure	Yes	30 (16)	37 (18)	>0.05
	No	156 (84)	169 (82)	

**Table 2:** Clinical symptoms of the patients before and after intervention.

	Before intervention			After intervention		
Variables	Intervention (*N*: 188)	Control (*N*: 208)	*P* value	Intervention (*N*: 188)	Control (*N*: 208)	*P* value
Otalgia	94 (50%)	106 (51%)	>0.05	28 (15%)	59 (28%)	<0.01^∗^
Fever	170 (90%)	172 (83%)	<0.01^∗^	35 (19%)	92 (44%)	<0.01^∗^
Irritability	86 (46%)	86 (41%)	>0.05	19 (10%)	40 (19%)	<0.05^∗^
Vomiting	6 (3%)	9 (4%)	>0.05	9 (5%)	4 (2%)	>0.05
Diarrhea	10 (5%)	8 (4%)	>0.05	13 (7%)	5 (2%)	<0.05^∗^
Coryza	107 (57%)	118 (57%)	>0.05	6 (3%)	10 (5%)	>0.05
Cough	92 (49%)	106 (51%)	>0.05	4 (2%)	10 (5%)	>0.05
Pharyngitis	4 (2%)	1 (0.5%)	>0.05	0 (0%)	0 (0%)	—
Nasal congestion	15 (8%)	10 (5%)	>0.05	1 (0.5%)	0 (0%)	>0.05
Skin rash	21 (11%)	25 (12%)	>0.05	2 (1%)	0 (0%)	>0.05
Asthma	7 (4%)	10 (5%)	>0.05	0 (0%)	0 (0%)	—
Poor feeding	4 (2%)	1 (0.5%)	>0.05	0 (0%)	0 (0%)	—
Ear drum perforation	x	x		2 (1%)	1 (0.5%)	>0.05
Unilateral erythema of TM	104 (55%)	129 (62%)	>0.05	^∗∗^	^∗∗^	
Bilateral erythema of TM	83 (44%)	78 (37%)	>0.05	^∗∗^	^∗∗^	
Unilateral MEE	93 (49%)	111 (53%)	>0.05	^∗∗^	^∗∗^	
Bilateral MEE	55 (29%)	59 (28%)	>0.05	^∗∗^	^∗∗^	

MEE: middle ear effusion; TM: tympanic membrane. ^∗^Statistically significant. ^∗∗^Not assessed after 3 days. X: ear drum perforation cases were excluded at initiation of the study.

**Table 3:** Association between treatment response and different factors.

		Intervention (*N*: 188)		Control (*N*: 208)		*P* value
		No	Yes	No	Yes	
Age	6-23 months	68 (74)	24 (26)	43 (44)	54 (56)	<0.01^∗^
	2-6 years	69 (72)	27 (28)	48 (43)	63 (57)	
Antibiotic in last 4 weeks	Yes	78 (72%)	31 (28%)	57 (42%)	79 (58%)	<0.01^∗^
	No	59 (75%)	20 (25%)	34 (47%)	38 (53%)	<0.01^∗^
Pneumococcal vaccination	Yes	3 (100%)	0 (0%)	1 (33%)	2 (67%)	>0.05
	No	114 (56%)	91 (44%)	50 (27%)	135 (73%)	<0.01^∗^
History of allergy	Yes	16 (64%)	9 (36%)	5 (24%)	16 (76%)	<0.01^∗^
	No	101 (55%)	82 (45%)	46 (27%)	121 (73%)	<0.01^∗^
History of asthma	Yes	8 (80%)	2 (20%)	1 (14%)	6 (86%)	<0.01^∗^
	No	109 (55%)	89 (45%)	50 (28%)	131 (72%)	<0.01^∗^
History of breastfeeding	Exclusive breastfeeding	76 (56%)	60 (44%)	32 (25%)	96 (75%)	<0.01^∗^
	Formula-fed	16 (57%)	12 (43%)	7 (29%)	17 (71%)	<0.05^∗^
	Both	25 (61%)	16 (39%)	12 (33%)	24 (67%)	<0.05^∗^
Child care type	Day care center	31 (54%)	26 (46%)	17 (35%)	31 (65%)	>0.05
	Babysitter	5 (62%)	3 (38%)	1 (10%)	9 (90%)	<0.05^∗^
	Mother	81 (57%)	62 (43%)	33 (25%)	97 (75%)	<0.01^∗^
Smoke exposure	Yes	20 (54%)	17 (46%)	10 (33%)	20 (67%)	>0.05
	No	95 (56%)	74 (44%)	39 (25%)	117 (75%)	<0.01^∗^
Erythema of TM	Unilateral	79 (76)	25 (24)	63 (49)	66 (51)	<0.01^∗^
	Bilateral	57 (69)	26 (31)	27 (35)	51 (65)	<0.01^∗^
MEE^∗∗^	Without effusion	32 (80)	8 (20)	26 (68)	12 (32)	>0.05
	Unilateral	70 (75)	23 (25)	47 (42)	64 (58)	<0.01^∗^
	Bilateral	35 (64)	20 (36)	18 (31)	41 (69)	<0.01^∗^

MEE: middle ear effusion; TM: tympanic membrane. ^∗^Statistically significant. ^∗∗^All patients did not refer for examination.

**Table 4:** Follow-up findings in patients after the intervention.

	Episode(s)	Intervention	Control	*P* value
AOM recurrence after 1 month	1	39 (21%)	50 (24%)	>0.05
	2	16 (8%)	16 (8%)	
	≥3	6 (3%)	4 (2%)	
AOM recurrence between 1 and 3 months	1	0 (0%)	5 (2%)	<0.05^∗^
	2	4 (2%)	14 (7%)	
	≥3	11 (6%)	9 (4%)	
MEE after 1 month		61 (32%)	70 (34%)	>0.05
MEE after 3 months		15 (8%)	28 (14%)	>0.05
Drug side effects	Total	20 (11%)	6 (3%)	<0.01^∗^
Type of side effects	Diarrhea	13 (7%)	5 (2%)	<0.05^∗^
	Vomiting	9 (5%)	4 (2%)	>0.05
	Skin rash	2 (1%)	0 (0%)	>0.05

MEE: middle ear effusion; AOM: acute otitis media. ^∗^Statistically significant.

**Table 5:** Association between AOM recurrence after 3 months and different factors.

		Intervention		Control		*P* value
		No	Yes	No	Yes	
Pneumococcal vaccination	Yes	1 (33)	2 (67)	1 (33)	2 (67)	>0.05
	No	61 (33)	124 (67)	75 (37)	130 (63)	>0.05
History of allergy	Yes	7 (33)	14 (67)	11 (44)	14 (56)	>0.05
	No	55 (33)	112 (67)	65 (35)	118 (65)	>0.05
History of asthma	Yes	2 (29)	5 (71)	2 (20)	8 (80)	>0.05
	No	60 (33)	121 (67)	74 (37)	124 (63)	>0.05
History of breastfeeding	Exclusive breastfeeding	41 (32)	87 (68)	48 (35)	88 (65)	>0.05
	Formula-fed	9 (37)	15 (63)	8 (29)	20 (71)	>0.05
	Both	12 (33)	24 (67)	18 (44)	23 (56)	>0.05
Child care type	Day care center	20 (42)	28 (58)	19 (33)	38 (67)	>0.05
	Babysitter	6 (60)	4 (40)	2 (25)	6 (75)	>0.05
	Mother	36 (28)	94 (72)	55 (38)	88 (62)	>0.05
Smoke exposure	Yes	9 (30)	21 (70)	14 (38)	23 (62)	>0.05
	No	52 (33)	104 (67)	61 (36)	108 (64)	>0.05

## Data Availability

The trial data used to support the findings of this study are available from the corresponding author upon request.
